# Cytomegalovirus Infection Modulates Cellular Immunity in an Experimental Model for Autoimmune Diabetes

**DOI:** 10.1080/10446670310001626490

**Published:** 2003

**Authors:** Nienke van der Werf, Jan-Luuk Hillebrands, Flip A. Klatter, Ineke Bos, Cathrien A. Bruggeman, Jan Rozing

**Affiliations:** 1 Faculty of Medical Sciences Department of Cell Biology, Immunology Section University of Groningen A. Deusinglaan 1 Groningen AV 9713 The Netherlands; 2 Department of Medical Microbiology University Hospital Maastricht P.O. Box 5800 Maastricht AZ 6202 The Netherlands

## Abstract

*Background*: Viral infections are thought to play a role in the development of autoimmune diseases like type 1 diabetes. In this study we investigated the effect of Rat Cytomegalovirus (RCMV) infection on cellular immunity in a well-defined animal model for diabetes, the Biobreeding (BB) rat.

*Methods*: Diabetes prone (DP)- and Diabetes resistant (DR)-BB rats were infected with 2 × 10^6^ plaque forming units (pfu) RCMV. Diabetes development was monitored by frequent blood-glucose analysis. Effects of RCMV on CD4^+^, CD8^+^ and Vβ-TCR^+^ T-cell subsets were measured *in vivo*, and *in vitro* after restimulation with RCMV-infected fibroblasts. Proliferative capacity was determined by ^3^H-Thymidine incorporation.

*Results*: RCMV-infection resulted in a significant acceleration of diabetes onset in DP-BB rats ( p=0.003). Percentages CD4^+^ and CD8^+^ T-cells were not affected *in vivo*. *In vitro*, RCMV-restimulation resulted in a decreased CD4^+^/CD8^+^ blastoid T-cell ratio compared to ConA ( p=0.00028). Furthermore, RCMV-restimulation resulted in a strong RCMV-specific proliferation, which comprises about 50% of the response triggered by ConA. Vβ-TCR percentages did not change upon RCMV-infection or RCMV-restimulation.

*Interpretation*: RCMV-restimulation of splenic T-cells *in vitro* resulted in a strong RCMV-specific proliferation, probably also including autoreactive T-cells. *In vivo*, this polyclonal response might be involved in the observed accelerated diabetes development in DP-BB rats upon RCMV-infection.


*Clinical & Developmental Immunology*, June–December 2003 Vol. 10 (2–4), pp. 153–160

Taylor & Francis healthsciences

### Cytomegalovirus Infection Modulates Cellular Immunity in an Experimental Model for Autoimmune Diabetes

NIENKE van der WERF[a], JAN-LUUK HILLEBRANDS[a], FLIP A. KLATTER[a], INEKE BOS[a], CATHRIEN A. BRUGGEMAN[b] and JAN ROZING[a,*]

[a]*Faculty of Medical Sciences, Department of Cell Biology, Immunology Section, University of Groningen, A. Deusinglaan 1, 9713 AV, Groningen, The Netherlands;* [b]*Department of Medical Microbiology, University Hospital Maastricht, P.O. Box 5800, 6202 AZ, Maastricht, The Netherlands*

*Background*: Viral infections are thought to play a role in the development of autoimmune diseases like type 1 diabetes. In this study we investigated the effect of Rat Cytomegalovirus (RCMV) infection on cellular immunity in a well-defined animal model for diabetes, the Biobreeding (BB) rat.

*Methods*: Diabetes prone (DP)- and Diabetes resistant (DR)-BB rats were infected with $2 \times 10^6$ plaque forming units (pfu) RCMV. Diabetes development was monitored by frequent blood-glucose analysis. Effects of RCMV on $CD4^+$, $CD8^+$ and $V\beta$-$TCR^+$ T-cell subsets were measured *in vivo*, and *in vitro* after restimulation with RCMV-infected fibroblasts. Proliferative capacity was determined by $^3$H-Thymidine incorporation.

*Results*: RCMV-infection resulted in a significant acceleration of diabetes onset in DP-BB rats ($p = 0.003$). Percentages $CD4^+$ and $CD8^+$ T-cells were not affected *in vivo*. *In vitro*, RCMV-restimulation resulted in a decreased $CD4^+/CD8^+$ blastoid T-cell ratio compared to ConA ($p = 0.00028$). Furthermore, RCMV-restimulation resulted in a strong RCMV-specific proliferation, which comprises about 50% of the response triggered by ConA. Vβ-TCR percentages did not change upon RCMV-infection or RCMV-restimulation.

*Interpretation*: RCMV-restimulation of splenic T-cells *in vitro* resulted in a strong RCMV-specific proliferation, probably also including autoreactive T-cells. *In vivo*, this polyclonal response might be involved in the observed accelerated diabetes development in DP-BB rats upon RCMV-infection.

*Keywords*: Autoimmunity; BB rats; Cytomegalovirus; Diabetes; Infection; Proliferation

### INTRODUCTION

Most autoimmune diseases (AID) have been shown to have a strong genetic component. However, studies on monozygous twins showed, that only in 50% of the cases both siblings developed an AID of the same type (McFarland, 1992; Kumar *et al.*, 1993). These data indicate that while AID occur in genetically susceptible individuals, many other factors must be involved in the phenotypic expression of autoimmunity (Rouse and Deshpande, 2002). Such non-genetic factors are environmental components, dietary constituents and infectious agents, particularly viruses. A variety of human viruses have been reported to be associated with AID. In Multiple Sclerosis (MS), one of the most common AID, both lesion induction and recurrence have been associated with many viruses (Whitton and Fujinami, 1999; Olson *et al.*, 2001). However, no particular virus has definitively been shown as the causative factor for MS disease. Autoimmune diabetes type 1 is another AID in which several viruses are suspected to play a role in the etiology, including CoxsackieB virus, rubella virus and mumps virus (Jun and Yoon, 2001). Another virus, which is thought to play a role in AID and in particular autoimmune diabetes, is Cytomegalovirus (CMV). This double stranded DNA virus, which is widely spread in the human population, causes a latent and persistent infection. In immunocompetent individuals primary CMV infections generally pass without clinical symptoms, whereas in immunocompromised hosts primary infections and reactivation of the virus cause severe morbidity and mortality (Plummer, 1973; Sinzger and Jahn, 1996). Several reports have suggested an association between CMV infection and autoimmunity. For instance, CMV infection is considered to be the most common viral antecedent infection of patients with Guillain–Barre syndrome (GBS). Jacobs *et al.* found a strong association of anti-GM2 IgM antibodies and CMV infection in GBS patients (Jacobs *et al.*, 1997). Since Ang *et al.* showed, that CMV infected human fibroblasts express the GM2 epitope (Ang *et al.*, 2000), these findings support the hypothesis of molecular mimicry between GM2 and CMV antigens as possible

*Corresponding author. Tel.: +31-50-3632530. Fax: +31-50-3632512. E-mail: j.rozing@med.rug.nl

ISSN 1740-2522 print/ISSN 1740-2530 online © 2003 Taylor & Francis Ltd
DOI: 10.1080/10446670310001626490

cause of GBS. Pak *et al.* showed a strong correlation between the presence of CMV genome and islet cell autoantibodies in newly diagnosed type 1 diabetic patients, suggesting an association between human type 1 diabetes and CMV infection (Pak *et al.*, 1988). Atherosclerosis is also associated with CMV infection. Adam *et al.* demonstrated in a clinical study that patients with high titres of CMV antibodies had a greater risk of developing clinically relevant atherosclerotic lesions (Adam *et al.*, 1987). In addition, CMV seropositive transplant patients are at substantially higher risk of suffering arterial occlusion, with subsequent graft loss or rejection, compared to CMV seronegative controls (Grattan *et al.*, 1989; Pouria *et al.*, 1998). Although there is a lot of data supporting a role for viruses such as CMV in autoimmune pathology, a causal relationship remains very difficult to demonstrate. One of the problems is that individuals might have encountered multiple viral infections at the time of diagnosis. Another problem is that immunological mediated damage might occur only after the viral infection has been cleared (von Herrath, 2000). Animal models could provide more direct evidence on the role of viral infections in AID. For instance, Semliki Forest Virus (SFV) infection of mice is one of the most useful experimental viral models of MS. SFV gives rise to an acute virus infection of the central nerve system (CNS), followed by demyelination. Mokhtarian *et al.*, (1999) reported that the virus exhibits molecular mimicry with a peptide of myelin oligodendrocyte glycoprotein (MOG). The antibody response against this epitope may well contribute to autoimmune mediated injury to CNS myelin.

The Diabetes prone (DP-BB) rat is a well-established model of autoimmune diabetes in rats (Crisa *et al.*, 1992). The animals develop autoimmune diabetes spontaneously at the age of 10–14 weeks. The diabetic syndrome, which in many aspects resembles human type 1 diabetes, results from the destruction of the pancreatic β cells by an autoimmune mediated process (Greiner *et al.*, 2001). Co-isogenic Diabetes resistant (DR) BB rats do not spontaneously develop autoimmune diabetes. However, when DR-BB rats are infected with Kilham Rat Virus (KRV), one-third of the infected animals developed autoimmune diabetes (Guberski *et al.*, 1991). Chung *et al.* showed that KRV-induced autoimmune diabetes in DR-BB rats is not due to molecular mimicry, but is due to a breakdown of the finely tuned immune balance resulting in the selective activation of β cell-cytotoxic effector T cells (Chung *et al.*, 2000).

In this study, we investigated the role of Rat Cytomegalovirus (RCMV) infection (e.g. rat equivalent of HCMV) in the DP-BB rat. Here we report that RCMV induces an accelerated onset of autoimmune diabetes in DP-BB rats. The mechanism by which RCMV infection induces autoimmune diabetes, however, is unknown. Previous studies showed that RCMV infection did not result in development of diabetes in a wide variety of rat strains, including the DR-BB rat. Together with the finding that the pancreatic islets are not infected upon RCMV infection, these data indicate that RCMV-induced lysis of the β cells is not likely to be the mechanism of RCMV induced diabetes. Another way in which RCMV might interfere in the diabetogenic process is by modulating T cell immunity. In this study, we investigated the cellular immune response against RCMV *in vivo* and *in vitro* to get more insight into the mechanism(s) through which RCMV modulates the autoimmune process in a rat model predisposed to develop autoimmune diabetes.

### RESULTS

### Diabetes Onset after RCMV Infection

In order to investigate whether RCMV influences the autoimmune process in DP-BB rats, animals were i.p. infected with $2 \times 10^6$ pfu RCMV at 35 days of age. As a result the mean diabetes onset was $67 \pm 13$ days in this group, which was significantly accelerated when compared to mock infected animals, which only developed diabetes at the age of $80 \pm 10$ days ($p = 0.003$). Figure 1 shows the accelerating effect of RCMV on the diabetes onset in DP-BB rats. In addition, the onset of insulitis (intra-islet lymphocytic infiltration) was also accelerated in RCMV infected rats (data not shown). In contrast, DR-BB rats which do not spontaneously develop diabetes, neither became diabetic, or developed insulitis after RCMV infection (data not shown).

### T Cell Subset Analysis *In Vivo*

The effect of RCMV infection on T cell subsets was analyzed by performing flow cytometric analysis on both spleen and PBMNC from DP and DR-BB rats after RCMV infection. Percentages of $CD4^+$ and $CD8^+$ T cell subsets are listed in Table I. DR-BB rats showed a small increase in the percentage of $CD8^+$ T cells 12 and 18 days after

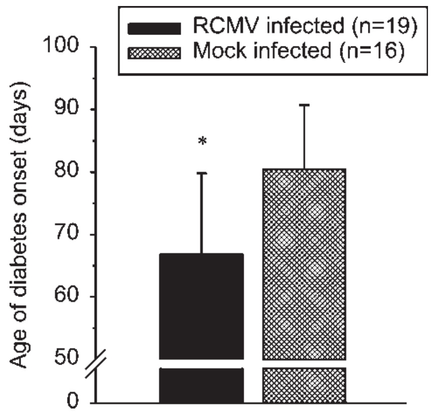

FIGURE 1 Mean time of onset of diabetes. DP-BB rats were infected with RCMV or mock infected at the age of 35 days and followed for the development of diabetes (see “Materials and methods” section). RCMV infection accelerated diabetes onset in DP-BB rats ($p = 0.003$).

TABLE I DR-BB rats show a small increase in the percentage of CD8+ T cells 12 and 18 days after RCMV infection in both spleen and peripheral blood

| | Spleen | | Peripheral blood | |
|---|---|---|---|---|
| | %CD4+ T-cells | %CD8+ T-cells | %CD4+ T-cells | %CD8+ T-cells |
| Untreated control | 57.9 ± 7.4* | 34.7 ± 4.2 | 72.8 ± 2.9 | 25.5 ± 2.7 |
| 6 days pi | 63.3 ± 0.7 | 31.4 ± 1.3 | 77.0 ± 2.1 | 21.1 ± 1.7 |
| 12 days pi | 53.3 ± 8.5 | 40.4 ± 7.3 | 67.3 ± 4.3 | 30.7 ± 4.4 |
| 18 days pi | 57.2 ± 6.5 | 38.6 ± 5.7 | 66.6 ± 6.8 | 31.8 ± 6.8 |

* Data are represented as mean ± SD. DR-BB rats were infected with $2.0 \times 10^6$ pfu RCMV at the age of 14 weeks. Both T cells from peripheral blood and splenocytes were isolated ($n = 3$/group) at 0, 6, 12 and 18 days after RCMV infection, labeled with FITC-conjugated OX8, PE-congugated OX35 and biotinylated R73 mAb coupled to Streptavidin Cychrome and analyzed on a flow cytometer.

infections in both spleen and PBMNC compared to uninfected controls (not significant). DP-BB rats showed a similar trend: the percentage of $CD8^+$ T cells is slightly increased 14 days after RCMV infection (data not shown). Taken together, RCMV infection resulted in a slightly increased $CD8^+$ T cell population *in vivo* in DP-BB as well as DR-BB rats.

### Proliferative Capacity of Splenocytes *In Vitro* after RCMV Restimulation

Furthermore, the *in vitro* proliferation capacity of DR splenocytes was examined by $^3$H-thymidine incorporation 18 days after RCMV infection. Since DP-BB splenic and peripheral blood lymphocytes have markedly defective proliferative responses in antigenic and mitogenic stimulation assays, DR splenocytes were used to determine *in vitro* proliferation upon RCMV stimulation (Elder and Maclaren, 1983; Georgiou *et al.*, 1988; Georgiou and Bellgrau, 1989). DR splenocytes show a strong RCMV specific proliferation (625 ± 163 dps) *in vitro* after restimulation with RCMV infected autologous fibroblasts, as compared to the unstimulated control (45 ± 14 dps). Since the RCMV specific response (625 ± 163 dps) comprises about 50% of the response triggered by Concanavalin A (1368 ± 14 dps), a polyclonal stimulator, these data suggest that over 50% of all T cells proliferated. This T cell response is RCMV specific because stimulation with uninfected fibroblasts did not induce such a strong proliferation (Fig. 2A). When splenocytes were isolated 35 days after infection and subsequently restimulated *in vitro*, a similar response was observed, although the RCMV specific response was less strong than 18 days after infection (data not shown). In addition, the *in vitro* stimulated DR splenocytes were labeled with mAb against TCRαβ and the percentage of blastocytes was determined by flow cytometric analysis. Blastocytes can phenotypically be identified as $TCR\alpha\beta^+$ and $FSC^{high}$. In accordance with the proliferation data as assessed by $^3$H-Thymidine incorporation, restimulation of RCMV infected splenocytes with autologous infected fibroblasts *in vitro* resulted in an increased blast transformation (41%) compared to splenocytes restimulated with uninfected fibroblasts (15%). Con A stimulation of both infected and uninfected splenocytes resulted in a 99% blastocyte transformation (Fig. 2B). These results confirmed that RCMV infection of DR-BB rats results in the generation of a strong proliferative T cell response, probably involving almost 50% of all T cells.

### T Cell Subset Analysis *In Vitro*

As shown in Table I RCMV infection did not induce major changes in the percentages of $CD4^+$ and $CD8^+$ T cells in DP-BB as well as in DR-BB rats *in vivo* and as a result the $CD4^+/CD8^+$ T cell ratio remained unaltered *in vivo* (Fig. 3A). We subsequently analyzed T cell subset distribution of DR-BB splenic T cells after restimulation *in vitro*. When DR splenic T cells were cultured for 4 days without stimulation (medium control or stimulation with uninfected Rat Embryonic Fibroblasts: $REF^-$) the $CD4^+/CD8^+$ T cell ratio increased (Fig. 3C), when compared with uncultured splenocytes isolated 22 days after RCMV infection (Fig. 3A; $p < 0.05$). Apparently, the percentage of $CD8^+$ T cells decreased when splenic T cells were cultured for 4 days in the absence of stimulatory signals. Con A stimulation did not alter the $CD4^+/CD8^+$ ratio within blastoid T cells when compared to unstimulated T cells. However, stimulation with autologous RCMV infected Rat Embryonic Fibroblasts ($REF^+$) resulted in a significantly lower $CD4^+/CD8^+$ ratio within splenic blastoid T cells when compared to ConA stimulation (Fig. 3B; $p = 0.00028$). These data thus show that RCMV restimulation of splenic T cells resulted in a decreased $CD4^+/CD8^+$ ratio within blastoid T cells, probably as a result of a preferential increase in the percentage of blastoid $CD8^+$ T cells after RCMV restimulation *in vitro*.

### Vβ Analysis of Splenic T Cells *In Vivo* and *In Vitro*

Data sofar showed that RCMV restimulation induced a strong RCMV specific proliferation of DR-BB splenic T cells *in vitro*. To test whether this response was superantigen-driven, a Vβ TCR analysis was performed on splenic T cells after *in vitro* stimulation. The percentages of three Vβ TCR families: Vβ16, Vβ10.2 and Vβ8.2 were measured by flow cytometric analysis. RCMV infection of DR-BB rats, however, did not affect the percentages of these Vβ TCR families within $CD4^+$

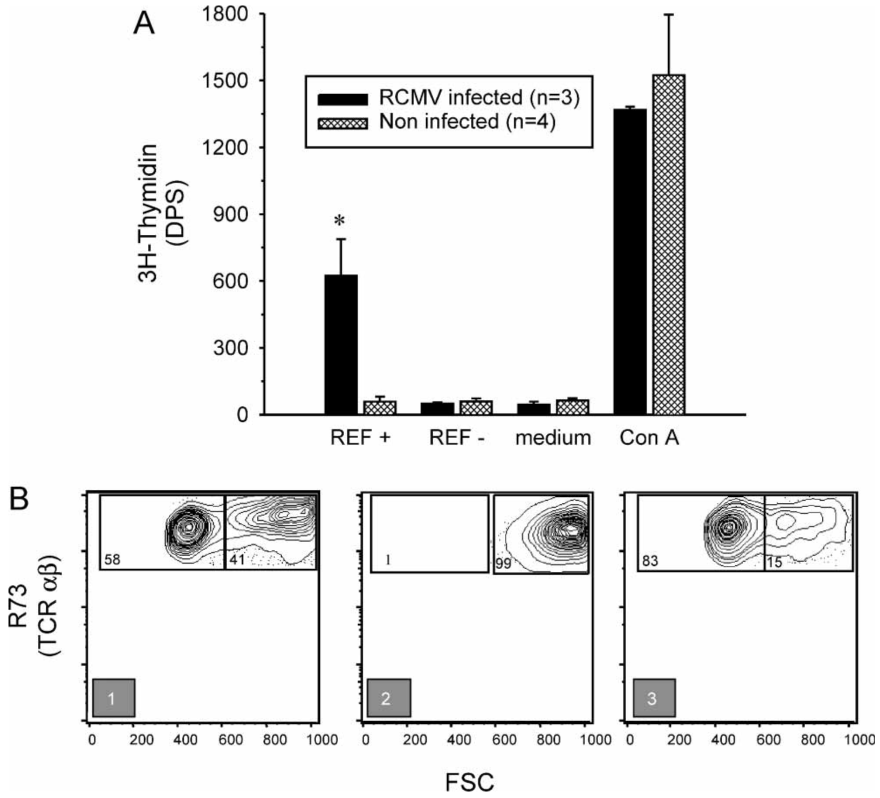

FIGURE 2 Proliferation and blast transformation of DR-BB splenic T cells *in vitro*. A: DR-BB splenocytes showed a strong RCMV specific proliferation *in vitro* after restimulation with infected autologous Rat Embryonal Fibroblasts ($REF^+$; $p < 0.05$)[1] B1-3: FACS plots[2] of a representative analysis showing blast transformation after restimulation *in vitro* with RCMV infected embryonic fibroblasts ($REF^+$, **B1**), ConA (**B2**) or non-infected fibroblasts ($REF^-$, **B3**). [1]DR spleens were removed from RCMV infected rats 18 days after infection and splenocytes were stimulated *in vitro* with infected or control Rat Embryonal Fibroblasts. As a positive control ConA stimulation (5 μg/ml) was used. Medium represents the unstimulated control. After 4 days of culture proliferation was determined by $^3$H-Thymidine incorporation. [2]Splenocytes were labeled with biotinylated R73 mAb coupled to Streptavidin Cychrome and the percentage of blastocytes was determined by gating the $R73^+$, $FSC^{high}$ T cells.

and $CD8^+$ T cell subsets when compared to uninfected controls *in vivo* (Fig. 4A,B). *In vitro* a similar pattern could be observed: percentages of the three Vβ TCR families did not alter after *in vitro* restimulation with RCMV infected fibroblasts (REF+) within the blastoid T cell population (REF+ versus ConA, Fig. 4C,D). In addition, the resting T cell population did neither show changes in Vβ TCR family distribution after *in vitro* restimulation with REF+ (REF+ versus REF−, Figure 4E,F). No changes could be observed between unstimulated splenocytes and splenocytes stimulated with control fibroblasts (REF−versus medium, data not shown).

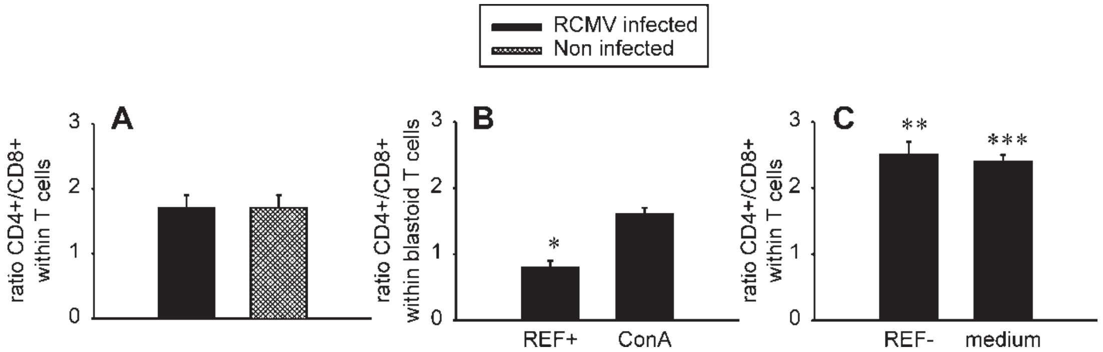

FIGURE 3 $CD4^+/CD8^+$ T cell ratio of splenic T cells. A: RCMV infection of DR-BB rats ($n = 3$/group) did not affect $CD4^+/CD8^+$ ratio in splenic T cells 22 days after infection compared to uninfected controls ($n = 2$/group)[1]. B: *In vitro* restimulation[2] of splenic T cells from RCMV infected DR-BB rats with RCMV infected Embryonic Fibroblasts ($REF^+$) ($n = 4$/group) resulted in a significant lower $CD4^+/CD8^+$ ratio within splenic blastoid T cells compared to ConA stimulation ($n = 3$/group) ($p = 0.00028$). C: *In vitro* restimulation[2] of splenic T cells from RCMV infected DR-BB rats with uninfected control Embryonic Fibroblasts ($REF^-$) ($n = 2$/group) or non-stimulated (medium) ($n = 4$/group) resulted in an increased $CD4^+/CD8^+$ T cell ratio compared to non-cultured splenic T cells (A; $p < 0.05$). [1]Splenocytes were labeled with FITC-conjugated OX8, PE-conjugated OX35 and biotinylated R73 mAb coupled to Streptavidin Cychrome and analyzed on a flow cytometer. [2]Splenocytes were isolated from RCMV infected DR-BB rats 18 days after infection and stimulated *in vitro* for 4 days with $REF^+$, $REF^-$, ConA (5 μg/ml) or left untreated.

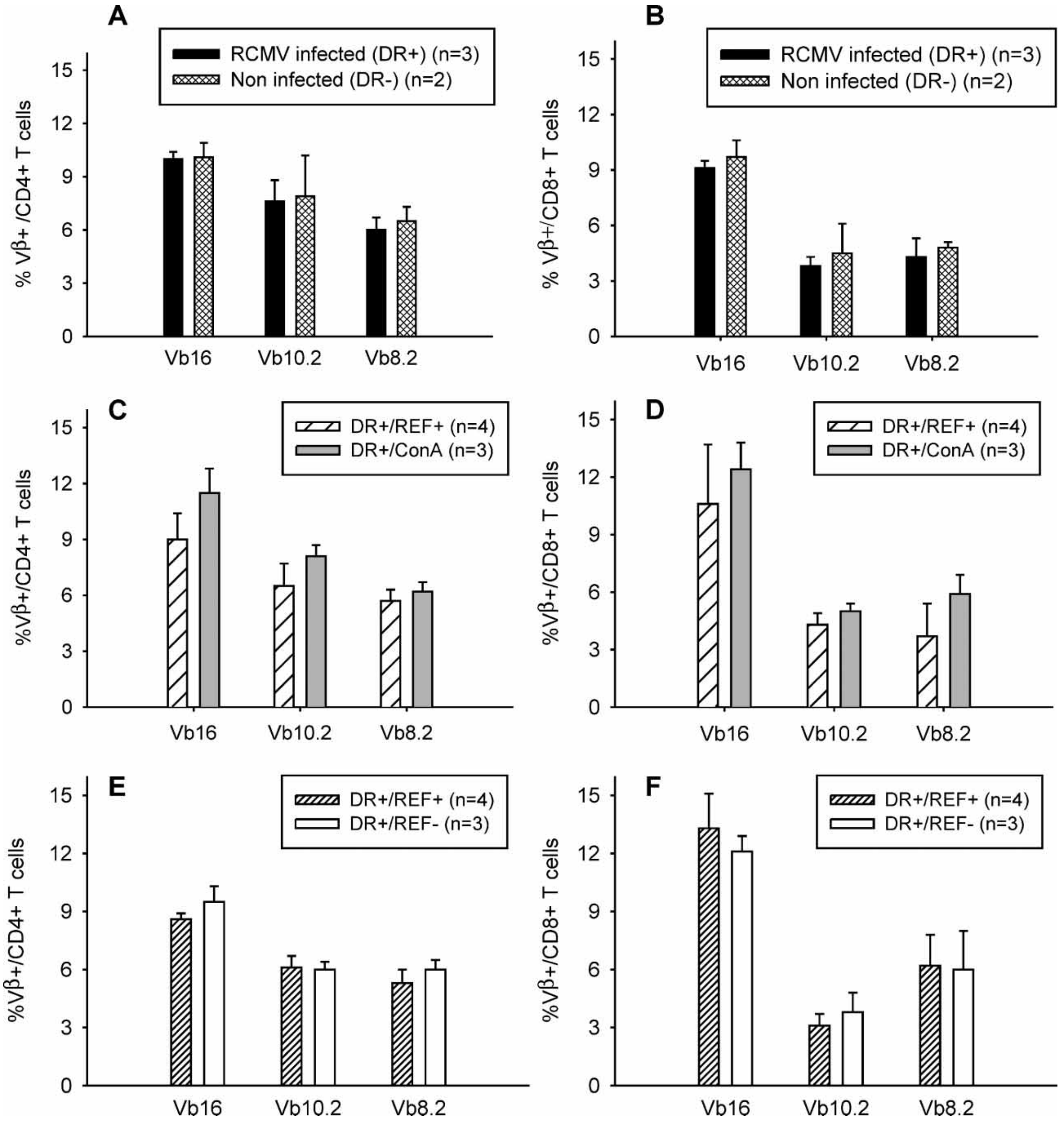

FIGURE 4 Analysis of three TCR Vβ families: Vβ16, Vβ10.2 and Vβ8.2 within splenic T cells of DR-BB rats. A-B: Vβ analysis of splenic T cells 22 days after RCMV infection[1]. C-F: Vβ analysis of splenic T cells of RCMV infected DR-BB rats restimulated *in vitro* with RCMV infected Rat Embryonic Fibroblasts ($REF^+$) or Con A (C,D) or restimulated *in vitro* with REF + or uninfected Rat Embryonic Fibroblasts ($REF^-$) (E,F)[1,2]. Percentage of $V\beta^+$ T cells were determined within the $CD4^+$ (A,C,E) or within the $CD8^+$ T cell subset (B,D,F) which is part of the blastoid T cell population (C,D) or resting T cells (E,F). [1]Splenocytes were labeled with FITC-conjugated Vβ16, Vβ10.2 or Vβ8.2, PE-conjugated OX35 (αCD4) and biotinylated R73 mAb coupled to Streptavidin Cychrome and analyzed on a flow cytometer. [2]Splenocytes were isolated from RCMV infected DR-BB rats 18 days after infection and stimulated *in vitro* for 4 days with $REF^+$, $REF^-$, ConA (5 μg/ml) or left untreated.

These data demonstrate that RCMV infection *in vivo* as well as *in vitro* does not result in Vβ TCR skewing for the three Vβ families tested.

### DISCUSSION

Environmental factors and in particular viral infections have been implicated in the etiology of various AID (Whitton and Fujinami, 1999; Olson *et al.*, 2001; Rouse and Deshpande, 2002). Autoimmune diabetes type 1 is such an AID in which several viral infections are suspected to play a role (Jun and Yoon, 2001). One of the candidate viral infections is CMV, a virus that is very common in the human population. Usually, a CMV infection passes without clinical symptoms. However, in immune compromised individuals reactivation can cause severe morbidity and mortality (Plummer, 1973; Sinzger and Jahn, 1996). Although there is a lot of data suggesting that viruses such as CMV play a role in AID, evidence for causal relationships is lacking. One of the problems is that the virus causing the disease might have been cleared from the patient at the time of diagnosis of AID (von Herrath, 2000). Animal models for AID could therefore provide more direct evidence on the role of viral infections in autoimmune pathology. The DP-BB rat, which spontaneously develops diabetes, is a well-characterized animal model for autoimmune diabetes. In this report we show that CMV infection accelerates the diabetogenic process in DP-BB rats. One of the possible underlying mechanisms of virus induced autoimmune diabetes could be direct lytic infection of the pancreatic β cells. This mechanism, however, seems not likely: PCR analysis showed the presence of RCMV genome in only a few islets isolated from infected BB rats (Hillebrands *et al.* this volume). Furthermore, CMV infection did not induce autoimmune diabetes in DR-BB rats, which are predisposed to develop diabetes but do not spontaneously develop autoimmune diabetes (Hillebrands *et al.* in preparation). One of the key factors is that DR-BB rats have regulatory $RT6^+$ T cells, whereas in DP-BB rats this regulatory population, which under normal conditions would prevent the autoimmunity to develop, is virtually absent (Crisa *et al.*, 1992; Mordes *et al.*, 1996). Another way in which RCMV might interfere in the diabetogenic process is by modulating T cell subsets. To test this hypothesis we investigated phenotype and proliferative capacity of the cellular immune response against RCMV *in vivo* and *in vitro* in BB rats. When DP-BB and DR-BB rats were infected with RCMV, a slight increase in the percentage of the $CD8^+$ T cell subset was found in DP-BB as well as in DR-BB rats at 18 days after viral infection. RCMV restimulation *in vitro* of splenic T cells resulted in a strong RCMV specific proliferation in which DNA synthesis comprises half of the mitotic events of polyclonal stimulated T cells. Indeed, FACS analysis of *in vitro* stimulated T cells showed that RCMV restimulation resulted in blast transformation of a significant part (41%) of the T cell population. Preliminary data of experiments, in which splenocytes were labeled with CFSE and subsequently stimulated *in vitro* and followed by FACS analysis, showed that approximately 8% of the T cell population entered mitosis after RCMV stimulation. The question arises whether RCMV acts here as a superantigen or a polyclonal stimulator. In the case of superantigenic stimulation RCMV peptide(s) could bind sequentially to MHC class II proteins and the Vβ chain of the T cell receptor, thereby activating and expanding predominantly $CD4^+$ T cells expressing the relevant Vβ family (Marrack *et al.*, 1993). Superantigens also elicit strong primary responses (Huber *et al.*, 1996).

Several reports indeed suggest a role for viral protein(s) with superantigenic activity in the development of autoimmune diabetes. Conrad *et al.* found a selective expansion of the TCR Vβ7 chain among islet infiltrating lymphocytes in two children with recent onset type 1 diabetes (Conrad *et al.*, 1994). Luppi *et al.* showed TCR Vβ7 skewing of the peripheral blood lymphocytes (PBL's) of recently-diagnosed diabetic patients compared to healthy individuals and patients with longterm diabetes, but also a temporal relationship between acute enterovirus infections and the increased percentage of Vβ7 gene transcripts (Luppi *et al.*, 2000). Stauffer *et al.* proposed that viral infections themselves might *induce* endogenous superantigens, like human endogenous retroviral superantigen $IDDMK_{1,2}22$ via IFNα production causing autoimmune disease like type 1 diabetes by activation of autoreactive T cells (Stauffer *et al.*, 2001). Dobrescu *et al.* furthermore showed that a CMV gene product was responsible for a superantigen-driven response: Vβ12-selective HIV1 replication was strongly promoted by direct contact with CMV infected monocytes in a way that is indistinguishable from the effect of known superantigens (Dobrescu *et al.*, 1995).

However, several of our data argue against a superantigenic nature of the strong *in vitro* RCMV responses we observed. Firstly, strong proliferative responses were only found after *in vitro* restimulation of *in vivo* primed splenic T cells. No such responses could be measured after primary stimulation with RCMV *in vivo*. Secondly, only expansion of $CD8^+$ T cells and not of $CD4^+$ T cells was seen after RCMV infection *in vivo* and *in vitro*. Thirdly, analysis of three Vβ $TCR^+$ T cell subsets indicated that RCMV infection did not result in specific Vβ skewing. These arguments rather support the view that RCMV induces a kind of polyclonal T cell response. What might then be the underlying mechanism of RCMV induced acceleration of the autoimmune process in DP-BB rats? We speculate that RCMV infection in diabetes prone individuals results in polyclonal T cell stimulation, thereby probably also activating and expanding existing autoreactive T cells. As a result destructive insulitis and consequently the development of diabetes is accelerated. On top of such a bystander activation of autoreactive cells also specific molecular mimicry between CMV epitopes and β cell antigens could be involved (Hiemstra *et al.*, 2001). Further experiments on the functional characteristics of this RCMV specific T cell response are needed to determine the pathogenic capacity of those RCMV activated T cells and their contribution to the eventual lysis of the pancreatic β cells, in order to get more insight in the role of the cellular immune response against RCMV in RCMV induced accelerated autoimmune diabetes in DP-BB rats.

In conclusion, RCMV restimulation of splenic T cells *in vitro* results in a strong RCMV specific proliferation, probably also including existing autoreactive T cells. *In vivo* such a polyclonal stimulation might be responsible for the accelerated destruction of pancreatic islets and finally, accelerated development of autoimmune diabetes in DP-BB rats.

### MATERIALS AND METHODS

### Animals

Diabetes Prone (DP-BB) and Diabetes Resistant (DR-BB) BB rats were bred at the central animal facility of the University of Groningen, The Netherlands. Original breeding stocks were provided by the University of Massachusetts, Worcester, MA, USA. The animals were raised under specific-pathogen-free (SPF) and viral-antibody-free (VAF) conditions and had water and food *ad libitum*. The University Ethical Board for Animal Studies approved all animal experiments reported in this study.

### Infection with RCMV

Rats of both sexes were injected intraperitoneally (i.p.) with $2 \times 10^6$ pfn of RCMV at the age of 35 days. The virus was isolated from salivary glands from infected rats as described previously (Maastricht strain) (Bruggeman *et al.*, 1982). Control rats were left untreated or mock infected. The latter was established by i.p. injection of 1 ml saline containing an isolate of salivary gland obtained from non-infected rats. Animals were observed twice a week for physical well being and checked for weight loss. When weight loss occurred, blood glucose levels were measured in tail vein blood using a Reflolux®S glucose sensor (Boehringer Mannheim, Germany). Diabetes was diagnosed when glucose levels exceeded 16 mM. Diabetic rats were killed at diagnosis and the remaining non-diabetic animals were killed at the age of 140 days.

### Stimulator Cells

Primary cultures of Rat Embryonic Fibroblasts (REF), which where produced partly according to the methods of Ross *et al.*, were used as stimulator cells (Ross *et al.*, 1996). REF were passaged twice and cultured in Minimal Earle's Medium (Gibco) supplemented with 1% Non Essential Amino Acids (Life technologies), 2 mM L-glutamin (Gibco), 100 μg/ml gentamycin and 20% Fetal Calf Serum (FCS) (Life technologies). When they had grown to confluence, the cells were serum-starved in two steps (10% resp. 2% FCS) and they were infected with RCMV for 24 h, with a multiplicity of infection of 1.5 pfu RCMV per cell. After 24 h culture medium was refreshed. After another 48 h the REF already showed significant signs of the RCMV induced cytopathic effects (cpe). When more than 90% of the cells showed cpe, they were harvested by adding PBS containing 1% trypsin and 0.5 mM EDTA. After washing, the REF were fixed in 1% paraformaldehyde for 10 min at room temperature ($1 \times 10^7$ cells/ml). After washing the cells once with 0.2 M glycine and once with culture medium, the REF were ready for use. Remaining REF were resuspended in Newborn Calf Serum (NCS) with 10% DMSO ($5 \times 10^5$ cells/ml) and stored at −80°C. Control stimulator cells were prepared in the same way except for the addition of virus.

### *In Vitro* Stimulation

Splenocytes were isolated from DP-BB and DR-BB rats at 18 and 35 days after RCMV infection. Briefly, spleens were dissected and pushed through an iron grid. After another, finer filtration step, erythrocytes in the cell suspension were lysed with $NH_4Cl$ for 5 min on ice. The splenocytes ($2 \times 10^5$ cells/well) were incubated for 4–6 days in 200 μl of complete RPMI 1640 medium containing 10% FCS, 5 mM sodium pyruvate, 5 mM L-glutamine, 0.05 mM 2-ME and 5 μg/ml gentamicin in 96 well roundbottom plates in the presence of RCMV infected rat embryonal fibroblasts ($REF^+$), uninfected REF ($REF^-$) at a responder:stimulator ratio of 10:1 or complete medium. REF are prepared as described under "stimulator cells". As a positive control, Concanavalin A (ConA) stimulation (5 μg/ml) was used. Splenocytes were harvested after a 4-day culture period for flow cytometric analysis and on days 4, 5 and 6 for $^3$H-Thymidine incorporation.

### *In Vitro* T Cell Proliferation Assay

On days 4, 5 and 6 after culture splenocytes were pulsed with 1 μCi $^3$H-Thymidine. After 16 h of incubation, the cells were harvested and the incorporated radioactivity was counted using a liquid scintillation counter.

### Flow Cytometry

Splenocytes and Peripheral Blood Mononuclear Cells (PBMNC) were isolated from DP-BB and DR-BB rats 18 and 35 days after RCMV infection. PBMNC were isolated from cardiac blood by spinning at a rate of 2500 rpm for 20 min after 1:1 dilution of the blood with Phosphate Buffered Saline (PBS). Buffycoats were taken and incubated with $NH_4Cl$ for erythrocyte lysis. PBMNC were resuspended in PBS containing 5% NCS, 0.5% Dulbecco B and 0.03% sodium azide (DAB/NCS/azide). Splenocytes were either freshly isolated or harvested after 4 days of *in vitro* stimulation as described above. The cells were incubated for 30 min at 4°C with FITC-conjugated OX-8 mAb (αCD8, Pharmingen), PE-conjugated OX35 mAb (αCD4, Pharmingen) and biotin-conjugated R73 mAb (αTCRαβ, Pharmingen) to determine $CD4^+$ and $CD8^+$ T cell subsets. After washing, Streptavidin-Cychrome (Pharmingen) was added to label biotin. Splenocytes and PBMNC isolated from uninfected rats were used as a control. To analyze Vβ TCR specific stimulation splenocytes were labeled with FITC-conjugated mAb's Vβ16, Vβ10.2 and Vβ8.2 (Pharmingen), PE-conjugated OX35 mAb and biotin-conjugated R73 mAb. After washing, Streptavidin-APC (Pharmingen) was added to label biotin. Cell populations were analyzed using an Epics Elite flow cytometer (Coulter Epics, Hialeah, USA).

### Statistical Analysis

Statistical analysis was performed using a two-tailed Student's *t* test. Values of $p < 0.05$ were considered to be significant.

### *Acknowledgements*

This study was supported by a grant of the Dutch Diabetes Foundation (DFN99.028).

### *References*

Adam, E., Melnick, J.L., Probtsfield, J.L., Petrie, B.L., Burek, J., Bailey, K.R., McCollum, C.H. and Debakey, M.E. (1987) "High levels of cytomegalovirus antibody in patients requiring vascular surgery for atherosclerosis", *Lancet* **2**, 291–293.

Ang, C.W., Jacobs, B.C., Brandenburg, A.H., Laman, J.D., van der Meche, F.G., Osterhaus, A.D. and van Doorn, P.A. (2000) "Cross-reactive antibodies against GM2 and CMV-infected fibroblasts in Guillain-Barre syndrome", *Neurology* **54**, 1453–1458.

Bruggeman, C.A., Meijer, H., Dormans, P.H., Debie, W.M., Grauls, G.E. and van Boven, C.P. (1982) "Isolation of a cytomegalovirus-like agent from wild rats", *Arch. Virol.* **73**, 231–241.

Chung, Y.H., Jun, H.S., Son, M., Bao, M., Bae, H.Y., Kang, Y. and Yoon, J.W. (2000) "Cellular and molecular mechanism for Kilham rat virus-induced autoimmune diabetes in DR-BB rats", *J. Immunol.* **165**, 2866–2876.

Conrad, B., Weidmann, E., Trucco, G., Rudert, W.A., Behboo, R., Ricordi, C., Rodriquez-Rilo, H., Finegold, D. and Trucco, M. (1994) "Evidence for superantigen involvement in insulin-dependent diabetes mellitus aetiology", *Nature* **371**, 351–355.

Crisa, L., Mordes, J.P. and Rossini, A.A. (1992) "Autoimmune diabetes mellitus in the BB rat", *Diabetes Metab. Rev.* **8**, 4–37.

Dobrescu, D., Ursea, B., Pope, M., Asch, A.S. and Posnett, D.N. (1995) "Enhanced HIV-1 replication in V beta 12 T cells due to human cytomegalovirus in monocytes: evidence for a putative herpesvirus superantigen", *Cell* **82**, 753–763.

Elder, M.E. and Maclaren, N.K. (1983) "Identification of profound peripheral T lymphocyte immunodeficiencies in the spontaneously diabetic BB rat", *J. Immunol.* **130**, 1723–1731.

Georgiou, H.M. and Bellgrau, D. (1989) "Thymus transplantation and disease prevention in the diabetes-prone Bio- Breeding rat", *J. Immunol.* **142**, 3400–3405.

Georgiou, H.M., Lagarde, A.C. and Bellgrau, D. (1988) "T cell dysfunction in the diabetes-prone BB rat. A role for thymic migrants that are not T cell precursors", *J. Exp. Med.* **167**, 132–148.

Grattan, M.T., Moreno-Cabral, C.E., Starnes, V.A., Oyer, P.E., Stinson, E.B. and Shumway, N.E. (1989) "Cytomegalovirus infection is associated with cardiac allograft rejection and atherosclerosis", *J. Am. Med. Assoc.* **261**, 3561–3566.

Greiner, D.L., Rossini, A.A. and Mordes, J.P. (2001) "Translating data from animal models into methods for preventing human autoimmune diabetes mellitus: caveat emptor and primum non nocere", *Clin. Immunol.* **100**, 134–143.

Guberski, D.L., Thomas, V.A., Shek, W.R., Like, A.A., Handler, E.S., Rossini, A.A., Wallace, J.E. and Welsh, R.M. (1991) "Induction of type I diabetes by Kilham's rat virus in diabetes- resistant BB/Wor rats", *Science* **254**, 1010–1013.

von Herrath, M.G. (2000) "Obstacles to identifying viruses that cause autoimmune disease", *J. Neuroimmunol.* **107**, 154–160.

Hiemstra, H.S., Schloot, N.C., van Veelen, P.A., Willemen, S.J., Franken, K.L., van Rood, J.J., de Vries, R.R., Chaudhuri, A., Behan, P.O., Drijfhout, J.W. and Roep, B.O. (2001) "Cytomegalovirus in autoimmunity: T cell crossreactivity to viral antigen and autoantigen glutamic acid decarboxylase", *Proc. Natl Acad. Sci. USA* **98**, 3988–3991.

Huber, B.T., Hsu, P.N. and Sutkowski, N. (1996) "Virus-encoded superantigens", *Microbiol. Rev.* **60**, 473–482.

Jacobs, B.C., van Doorn, P.A., Groeneveld, J.H., Tio-Gillen, A.P. and van der Meche, F.G. (1997) "Cytomegalovirus infections and anti-GM2 antibodies in Guillain-Barre syndrome", *J. Neurol. Neurosurg. Psychiat.* **62**, 641–643.

Jun, H.S. and Yoon, J.W. (2001) "The role of viruses in type I diabetes: two distinct cellular and molecular pathogenic mechanisms of virus-induced diabetes in animals", *Diabetologia* **44**, 271–285.

Kumar, D., Gemayel, N.S., Deapen, D., Kapadia, D., Yamashita, P.H., Lee, M., Dwyer, J.H., Roy-Burman, P., Bray, G.A. and Mack, T.M. (1993) "North-American twins with IDDM. Genetic, etiological, and clinical significance of disease concordance according to age, zygosity, and the interval after diagnosis in first twin", *Diabetes* **42**, 1351–1363.

Luppi, P., Zanone, M.M., Hyoty, H., Rudert, W.A., Haluszczak, C., Alexander, A.M., Bertera, S., Becker, D. and Trucco, M. (2000) "Restricted TCR V beta gene expression and enterovirus infection in type I diabetes: a pilot study", *Diabetologia* **43**, 1484–1497.

Marrack, P., Winslow, G.M., Choi, Y., Scherer, M., Pullen, A., White, J. and Kappler, J.W. (1993) "The bacterial and mouse mammary tumor virus superantigens; two different families of proteins with the same functions", *Immunol. Rev.* **131**, 79–92.

McFarland, H.F. (1992) "Twin studies and multiple sclerosis", *Ann. Neurol.* **32**, 722–723.

Mokhtarian, F., Zhang, Z., Shi, Y., Gonzales, E. and Sobel, R.A. (1999) "Molecular mimicry between a viral peptide and a myelin oligodendrocyte glycoprotein peptide induces autoimmune demyelinating disease in mice", *J. Neuroimmunol.* **95**, 43–54.

Mordes, J.P., Bortell, R., Doukas, J., Rigby, M., Whalen, B., Zipris, D., Greiner, D.L. and Rossini, A.A. (1996) "The BB/Wor rat and the balance hypothesis of autoimmunity", *Diabetes Metab. Rev.* **12**, 103–109.

Olson, J.K., Croxford, J.L. and Miller, S.D. (2001) "Virus-induced autoimmunity: potential role of viruses in initiation, perpetuation, and progression of T-cell-mediated autoimmune disease", *Viral Immunol.* **14**, 227–250.

Pak, C.Y., Eun, H.M., McArthur, R.G. and Yoon, J.W. (1988) "Association of cytomegalovirus infection with autoimmune type 1 diabetes", *Lancet* **2**, 1–4.

Plummer, G. (1973) "Cytomegaloviruses of man and animals", *Prog. Med. Virol.* **15**, 92–125.

Pouria, S., State, O.I., Wong, W. and Hendry, B.M. (1998) "CMV infection is associated with transplant renal artery stenosis", *QJM* **91**, 185–189.

Ross, P.S., van Loveren, H., de Swart, R.L., van der Vliet, H., de Klerk, A., Timmerman, H.H., van Binnendijk, R., Brouwer, A., Vos, J.G. and Osterhaus, A.D. (1996) "Host resistance to rat cytomegalovirus (RCMV) and immune function in adult PVG rats fed herring from the contaminated Baltic Sea", *Arch. Toxicol.* **70**, 661–671.

Rouse, B.T. and Deshpande, S. (2002) "Viruses and autoimmunity: an affair but not a marriage contract", *Rev. Med. Virol.* **12**, 107–113.

Sinzger, C. and Jahn, G. (1996) "Human cytomegalovirus cell tropism and pathogenesis", *Intervirology* **39**, 302–319.

Stauffer, Y., Marguerat, S., Meylan, F., Ucla, C., Sutkowski, N., Huber, B., Pelet, T. and Conrad, B. (2001) "Interferon-alpha-induced endogenous superantigen. A model linking environment and autoimmunity", *Immunity* **15**, 591–601.

Whitton, J.L. and Fujinami, R.S. (1999) "Viruses as triggers of autoimmunity: facts and fantasies", *Curr. Opin. Microbiol.* **2**, 392–397.

